# Pulp response following direct pulp capping with Tideglusib and mineral trioxide aggregate: an animal study

**DOI:** 10.1186/s12903-025-06546-6

**Published:** 2025-07-19

**Authors:** Momen M. Mhmod, Ehab E. Hassanien, Ashraf M. Abu-Seida, Salma H. EL Ashry, Mohamed M. Nagy, Sara H. Fahmy, Elhassan E.E. Hassanein

**Affiliations:** 1 Endodontic Department, Faculty of Dentistry, October University for Modern Sciences & Arts, Giza, Egypt; 2 Endodontic Department, Faculty of Dentistry, Galala University, Suez, Egypt; 3 Endodontic Department, Faculty of Dentistry, Ain Shams University, Cairo, Egypt; 4 Department of Surgery, Anesthesiology & Radiology, Faculty of Veterinary Medicine, Cairo University, PO: 12211, Giza, Egypt; 5 HHU Heinrich Heine Universität Düsseldorf, Düsseldorf, Germany

**Keywords:** Dentin repair, Mineral trioxide aggregate, Pulp inflammation, Tideglusib, Vital pulp therapy, Wnt/β-catenin signaling pathway

## Abstract

**Background:**

Several biomaterials have been employed for direct pulp capping (DPC) with varying degrees of success. This study evaluated the pulp response following DPC with a new material developed from glycogen synthase kinase-3 inhibitors (Tideglusib) and mineral trioxide aggregate (MTA).

**Methods:**

Class V cavities with pulp exposure were conducted on 56 teeth in two adult male mongrel dogs. Based on the evaluation periods, these teeth were divided into two major groups at random (28 teeth/dog each). Groups A and B underwent histopathology evaluations three and eight weeks following DPC, respectively. Depending on the capping material used, each main group was further divided into two equal subgroups (14 teeth each). ProRoot white MTA was applied directly to the exposed vital pulps in subgroup 1. While subgroup 2’s exposed pulps were immediately capped with resorbable collagen that had been soaked in a freshly made 50 nM Tideglusib drug solution. Glass ionomer filling was then used to seal the access cavities. Every specimen underwent histological evaluation and was scored according to the number of inflammatory cells, the disorganization of the pulp tissue, and the formation of calcific bridges. All data were statistically examined.

**Results:**

In both groups A and B, subgroup 2 showed a statistically significant increase in the number of inflammatory cells and pulp tissue disorganization compared to subgroup 1 (*P* < 0.05). In both groups A and B, there was no statistically significant difference in the formation of new hard tissue between subgroups 1 and 2 (*P* = 0.157).

**Conclusion:**

When used as direct vital pulp capping materials in a dog model, Tideglusib causes more soft tissue disorganization and an inflammatory response inside the pulp cavity than ProRoot white MTA.

## Background

The creation of new biocompatible materials, along with a better understanding of the dentine-pulp complex’s ability to heal, has led to a rise in interest in vital pulp therapy over the past decade [1–3]. These advancements have enhanced the reliability and potential of vital pulp tissue preservation. Instead of complete pulpectomy, vital pulp therapies are currently endorsed by position statements from both the American Association of Endodontists and the European Society of Endodontists [4].

Direct pulp capping (DPC) is a noninvasive and effective procedure that entails placing a biocompatible material on the exposed pulp tissue to seal it and stimulate the stem cells within the pulp to differentiate into odontoblast-like cells, promoting the formation of reparative dentine [5]. To ensure that exposed dental pulp heals correctly, an ideal pulp capping material must be bioactive, biocompatible, biostable, and retain antimicrobial properties against different microorganisms. Various DPC materials, including calcium hydroxide, MTA, and other calcium silicate cements, have succeeded in biological healing. Even though MTA is regarded as the gold standard DPC material, the majority of these materials don’t meet al.l of the previously listed requirements [1, 3, 5].

Following DPC, numerous materials analyzed in the literature have demonstrated improved wound healing and reparative dentin formation [6–12]. Restoring the normal dentine volume entirely and effectively is unattainable due to the insolubility of these materials. To substitute the lost dentine in a manner that promotes the natural mechanism of reparative dentine development by drawing in local undifferentiated stem cells from the dental pulp, new biologically acceptable methods need to be created.

Glycogen synthase kinase-3 (GSK-3) is a serine/threonine kinase found in all eukaryotic organisms and regulates several pathways, including the Hedgehog pathway, which communicates signals to embryonic cells for proper cell differentiation, and the Wnt/β-catenin signaling pathway, essential for maintaining cellular homeostasis [13]. GSK-3 inhibitors, capable of speeding up the synthesis of reparative dentine to completely regenerate the harmed hard tissue, were first employed in biodegradable collagen sponges [14].

After tissue injury, the Wnt/β-catenin signaling pathway is activated, which is crucial for drawing in mesenchymal stem cells (MSCs) and altering the formation of reparative dentine. Several approaches can be employed to trigger this system. A crucial cytoplasmic element of this system, GSK-3 adds phosphate groups to β-catenin and Axin, leading to their degradation when there is no interaction between Wnt ligands and receptors. Wnt ligands restrict the function of the GSK-3 enzyme, allowing β-catenin to move into the nucleus and activate target genes, including Axin2. Given that it has been confirmed that dental injury triggers Axin2 expression and Wnt/β-cat signaling, the inclusion of GSK-3 inhibitors or Wnt/β-catenin agonists could promote the development of reparative dentine [15, 16].

Several GSK-3 inhibitor medications include Tivantinib, Tideglusib, and Cromolyn. They possess the ability to induce reparative dentine by activating Wnt signaling activity [14, 17, 18]. To our knowledge, there are only a limited number of studies that outline the histological assessment of pulp tissue after DPC using GSK-3 antagonists such as Tideglusib. As a result, this research compared the impact of Tideglusib and MTA when used as DPC materials in a dog model. The study’s null hypothesis was that there would be no statistically significant differences in the number of inflammatory cells, pulp tissue disorganization, or hard tissue formation between MTA and Tideglusib as DPC agents.

## Methods

### Ethical approval

This research received approval from the Ethical Committee at the Faculty of Dentistry, Ain Shams University, Egypt (Approval number: 105 − 15/07/2020). The research adhered to the five freedoms that define the key elements of animal welfare under human oversight, which include freedom from pain, hunger or thirst, discomfort, the ability to express normal behavior, and fear as well as distress. Additionally, the current study adhered to the Animal Research: Reporting in Vivo Experiments (ARRIVE) guidelines.

### Animals’ selection and preparation

This study involved two healthy adult male mongrel dogs that had a full set of intact, healthy permanent teeth. The dogs were acquired through commercial means from Al-Fahad Trading Company for Animals (Abu-Rawash, Giza, Egypt). The participating dogs had an average weight of 20 kg and a mean age of two years. Every animal was kept in an individual kennel within the animal house.

Before the experiment, dogs underwent clinical and radiographic examinations to confirm a healthy periodontium. Any dog exhibiting wounds, systemic illness, fractures, infections, or carious lesions was discarded from the study. Food was provided twice a day, and clean, pure water was accessible *ad libitum* for both dogs.

### Classifications of samples

Two dogs, with a combined total of 56 teeth, were randomly divided (block randomization) into two primary groups (28 teeth/one dog each), according to the assessment periods. The histopathology assessment was performed following 3 and 8 weeks of DPC in groups A and B, respectively.

Each primary group was additionally divided into two equal subgroups (14 teeth each) based on the type of capping material employed. In subgroup 1, the experimentally exposed vital pulp was directly capped with Pro-root white MTA (ProRoot MTA^®^, Dentsply Tulsa Dental, USA). In subgroup 2, the exposed vital pulp was directly capped with resorbable collagen (Type I intact collagen sponge^®^, Eucare Pharmaceuticals, India) that had been soaked in a freshly prepared solution of 50 nM Tideglusib drug (Tideglusib^®^, Sigma Aldrich, USA). Both subgroups were represented in each dog.

### Preparation of Tideglusib drug

Clinically validated biodegradable collagen sponges were utilized to administer the drug directly to the exposed pulp after being immersed in the freshly made Tideglusib solution. The appropriate quantity of Tideglusib powder was calculated using its molecular weight (334.39 g/mol) and its solubility in dimethyl sulfoxide (DMSO) solvent (> 15 mg/mL). Tideglusib was subsequently dissolved in a DMSO solution to achieve the target concentration of 30 nM/mL.

### Surgical procedure

At the Faculty of Veterinary Medicine, Cairo University, a proficient veterinarian offered diligent oversight throughout each animal’s anesthesia process. Following a 12-hour fast, 0.05 mg/kg of body weight of subcutaneous atropine sulfate (Atropine sulphate^®^, ADWIA Co., Egypt) and 1 mg/kg of body weight of intramuscular xylazine HCl (Xylaject 2%^®^, ADWIA Co., Egypt) were administered to the animal for premedication. Ketamine HCl (Keiran^®^, EIMC Pharmaceuticals Co., Egypt) was given intravenously at a dose of 10 mg/kg body weight to initiate general anesthesia, which was maintained during the procedure using additional doses of a 2.5% thiopental sodium solution (Thiopental sodium^®^, EIPICO, Egypt) at 25 mg/kg.

Under the guidance of a specialist with thirty years of experience in experimental dentistry (Prof. Ashraf Abu-Seida, at Cairo University’s Faculty of Veterinary Medicine), the same endodontist (Dr. Momen M. Mhmod) who had previously received training performed all the subsequent surgeries. The teeth of the dog (second and third incisors, canines, and premolars) were cleaned, scaled with ultrasound, and polished using a rubber cup prior to cavity preparation [19]. The teeth were treated with a 0.5% povidone iodine solution (Betadine^®^, Nile Company, Egypt) for disinfection.

Class V cavities, measuring 1–2 mm above the gingival margins, were created with continuous coolant and abundant saline irrigation on the buccal surfaces of the teeth involved, parallel to the cemento-enamel junction (CEJ). The depth of the cavity floor was increased until the shadow of the pulp tissue became visible, and a new 0.5 mm diameter carbide bur was used for cavity preparation to prevent thermal injury to the pulp. A sterile sharp endodontic probe was utilized to intentionally create a mechanical exposure, minimizing excessive harm to the pulp tissue [8, 10]. Bleeding was managed through continuous sterile saline irrigation until natural hemostasis was achieved, then dried with sterile cotton pellets [5, 6].

In subgroups A1 and B1, MTA was combined with a sterile spatula on a sterile glass slab following the manufacturer’s guidelines to achieve the required consistency. The mixture was subsequently applied to the exposure site with a carrier and then gently condensed using a moistened cotton pellet to guarantee suitable adaptation on the exposed vital pulp tissues [6].

In subgroups A2 and B2, the exposed vital pulp was capped with an absorbable collagen membrane that had been soaked in 30 nM Tideglusib dissolved and diluted in DMSO solvent [14]. The glass ionomer filling (Medifill^®^, Promedica, Germany) was used to seal the access cavities.

Dogs were fed a soft, balanced diet throughout the experiment. Carprofen tablets (Rimadyl Chewable Tablets^®^, Zoetis, USA) were given orally for 5 days at a dose of 4.4 mg/kg/ once daily as a postoperative analgesic. Additionally, 10 mg/kg body weight of cefotaxime (Cefotax^®^, EPICO, Egypt) was administered intramuscularly as antibiotic to both dogs for five days.

### Histological evaluation

At the end of every experimental phase, the dogs were euthanized after three and eight weeks via a rapid injection of 20 ml of 5% thiopental sodium through the cephalic vein [8, 10]. Maxillae and mandibles were cut and divided with a saw into two parts. The remains of the animal body were incinerated in the medical waste facility at the Faculty of Veterinary Medicine, Cairo University.

Following resection, each experimental tooth was cut for reduced decalcification duration. Samples were preserved in 10% neutral buffered formalin. The specimens were decalcified by being immersed in formic acid for two consecutive weeks, followed by an EDTA solution for four months. Throughout this time, the decalcifying solution was replaced with a new mixture every 2 days [6]. The decalcified samples were handled using an open processing system, where they were dehydrated in various increasing concentrations of ethyl alcohol: 70%, 95%, and absolute alcohol over a period of eighteen hours. The samples were embedded in paraffin wax, and sections from each block were sliced with a microtome at 4 to 5 microns thick in the buccolingual direction relative to the vertical axis through the capping site and pulp tissue [5, 6, 10]. All samples were assessed and rated based on the count of inflammatory cells, the formation of calcific bridges, and the disorganization of pulp tissue.

#### Assessment of inflammatory cells count

All regions displaying pulp tissue and capping material were recorded using a digital camera (EOS 650D, Canon, Japan) that was attached to the light microscope (BX60, Olympus, Japan). The images that were captured were subsequently moved to the computer system for analysis. The histomorphometric evaluation was conducted utilizing Image J analysis software (1.41a, NIH, USA). For every slide, four microscopic fields were assessed at 40x magnification. Chosen areas validated the subsequent criteria: infiltration of inflammatory cells, well-preserved tissue exhibiting good architecture, and absence of artifacts.

The dimensions of inflammatory cells were assessed, yielding a size range in pixel 2 and corresponding circularity measurements. The picture of all inflammatory cells of varying sizes was transformed into an 8-bit grayscale format. The threshold for color coding was adjusted to select the boundary of inflammatory cells to eliminate other unwanted structures. Subsequently, the binary thresholds for the identified color-coded inflammatory cells were modified prior to the calculation. A threshold was applied to the image, and the cells within the specified range of size and circularity were counted for both the smallest and largest inflammatory cells.

#### Assessment of pulp tissue disorganization

The disorganization of pulp tissue was evaluated based on the criteria set by Dogheim et al. [20] as outlined below:

Score 0: normal or near-normal tissue structure.

Score 1: disorganized odontoblastic layer surrounded by normal pulp in the center.

Score 2: complete disorganization of pulp tissue.

Score 3: pulp necrosis.

#### Evaluation of new hard tissue development

Sections were observed under a microscope at 40x magnification to identify calcific bridges [6] as detailed below:

Scores of 0, 1, and 2 indicated no, partial, and full new hard tissue formation, respectively [21].

### Statistical analysis

The mean and standard deviation values were determined for every evaluation within each group. The normality of the data was examined through the Kolmogorov-Smirnov and Shapiro-Wilk tests; pulp tissue disorganization and hard tissue formation data exhibited a non-parametric distribution, whereas the other data indicated a parametric distribution. The Mann Whitney test was employed to compare two groups in unrelated samples for non-parametric data.

The Wilcoxon test was utilized to compare the two groups in related samples. An independent sample *t*-test was employed to compare the two groups in unrelated samples. A paired sample *t*-test was conducted to compare the two groups in related samples. The significance level was established at *P* ≤ 0.05. Statistical examination was conducted using IBM^®^ SPSS^®^ Statistics Version 20 for Windows.

## Results

### Results of inflammatory cells count

Statistically significant differences were observed in the inflammatory cell counts between subgroup A1 and subgroup A2 (*P* < 0.001) as well as between subgroup B1 and subgroup B2 (*P* < 0.001) as shown in Table 1. The greatest mean counts of inflammatory cells were observed in subgroups A2 and B2, while the lowest mean values were seen in subgroups A1 and B1.

**Table 1:** Mean, standard deviation and range values of inflammatory cells count, pulp tissue disorganization and hard tissue formation

Capping agents (Subgroups)	Evaluation periods (Groups)		*P*- value
	Group A (After 3 weeks)	Group B (After 8 weeks)	
	Inflammatory cells count		
	Mean ± SD (Range)	Mean ± SD (Range)	
**Subgroup 1 (MTA)**	86.23 ± 1.05 (70–97)	0.00 ± 0.00	< 0.001*
**Subgroup 2 (Tideglusib)**	125.43 ± 8.96 (66–236)	224.00 ± 10.78 (120–334)	< 0.001*
***P- value***	< 0.001*	< 0.001*	
**Pulp tissue disorganization**			
**Subgroup 1 (MTA)**	1.05 ± 0.23 (1–3)	0.71 ± 0.16 (0–2)	0.011*
**Subgroup 2 (Tideglusib)**	2.14 ± 0.18 (1–3)	1.50 ± 0.20 (1–3)	0.021*
***P- value***	0.013*	0.018*	
**Hard tissue formation**			
**Subgroup 1 (MTA)**	0.00 ± 0.00	0.14 ± 0.10	0.150ns
**Subgroup 2 (Tideglusib)**	0.00 ± 0.00	0.00 ± 0.00	0.317ns
***P- value***	0.157ns	0.157ns	

*: Significant at *P* < 0.05

Concerning the assessment period, there were statistically significant differences between subgroups A1 and B1 as well as between subgroups A2 and B2 (*P* < 0.001). The maximum mean inflammatory cell count was observed in subgroup B2 (224.00 ± 10.78), while the minimum mean value was noted in subgroup B1 (0.00).

### Results of pulp tissue disorganization

There were statistically significant differences in pulp tissue disorganization between subgroups A1 and A2 (*P* = 0.013) and between subgroups B1 and B2 (*P* = 0.018) as shown in Table 1. The greatest mean value of pulp tissue disorganization was observed in subgroup B2, whereas the lowest mean value was noted in subgroup A1. The distribution of samples (*n* = 14) among the scores of pulp tissue disorganization in all subgroups is shown in Table 2.

**Table 2:** Distributions of samples (*n* = 14) among the scores of pulp tissue disorganization and new hard tissue formation in different subgroups

Pulp tissue disorganization				
Groups/ subgroups	Score 0	Score 1	Score 2	Score 3
**Subgroup A1 (MTA after 3 weeks)**	0	10	1	3
**Subgroup A2 (Tideglusib after 3 weeks)**	0	2	8	4
**Subgroup B1 (MTA after 8 weeks)**	5	8	1	0
**Subgroup B2 (Tideglusib after 8 weeks)**	0	9	3	2
**Hard tissue formation**				
**Groups/ subgroups**	**Score 0**	**Score 1**	**Score 2**	
**Subgroup A1 (MTA after 3 weeks)**	14	0	0	
**Subgroup A2 (Tideglusib after 3 weeks)**	14	0	0	
**Subgroup B1 (MTA after 8 weeks)**	12	2	0	
**Subgroup B2 (Tideglusib after 8 weeks)**	14	0	0	

Concerning the evaluation duration, statistically significant differences were observed between subgroups A1 and B1 (*P* = 0.011) as well as between subgroups A2 and B2 (*P* = 0.021). The greatest mean value was identified in subgroup A2, whereas the lowest mean value was discovered in subgroup B1.

### Results of new hard tissue formation

There were no statistically significant differences in new hard tissue formation between subgroups A1 and A2 as well as between subgroups B1 and B2 (*P* = 0.157) as shown in Table 1. The mean hard tissue formation scores for subgroups A1 and A2 were 00.00. The mean hard tissue formation scores for subgroups B1 and B2 were 0.14 ± 0.10 and 00.00, respectively. The distribution of samples (*n* = 14) among the scores of new hard tissue formation in all subgroups is shown in Table 2.

**Fig. 1:**
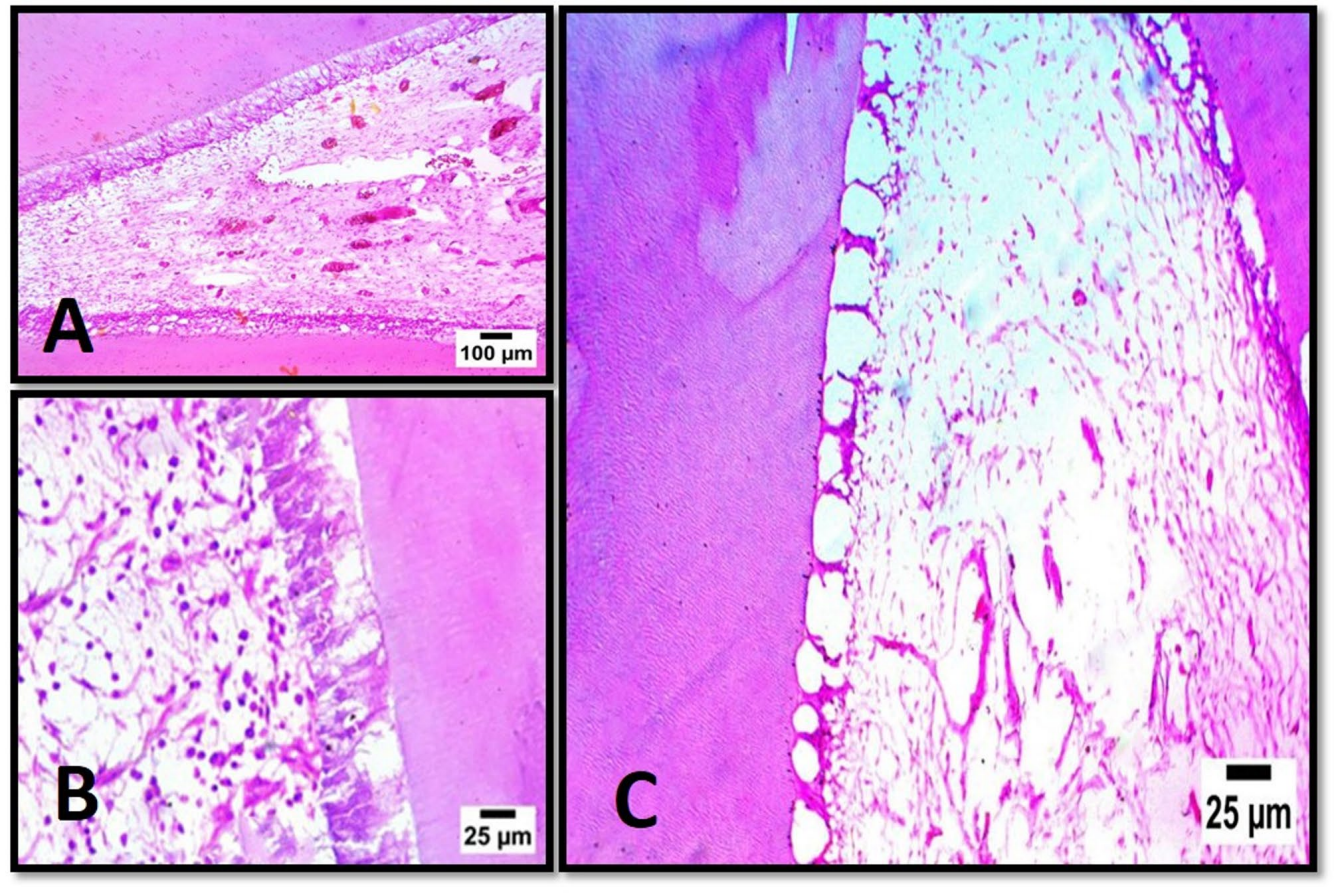
Representative photomicrographs of different subgroups showing noticeable angiogenic activity in subgroup B1 (**A**), noticeable inflammatory cells infiltration in subgroup B2 (**B**), and disorganized pulp tissue in subgroup A2 (**C**). (H&E stain)

**Fig. 2:**
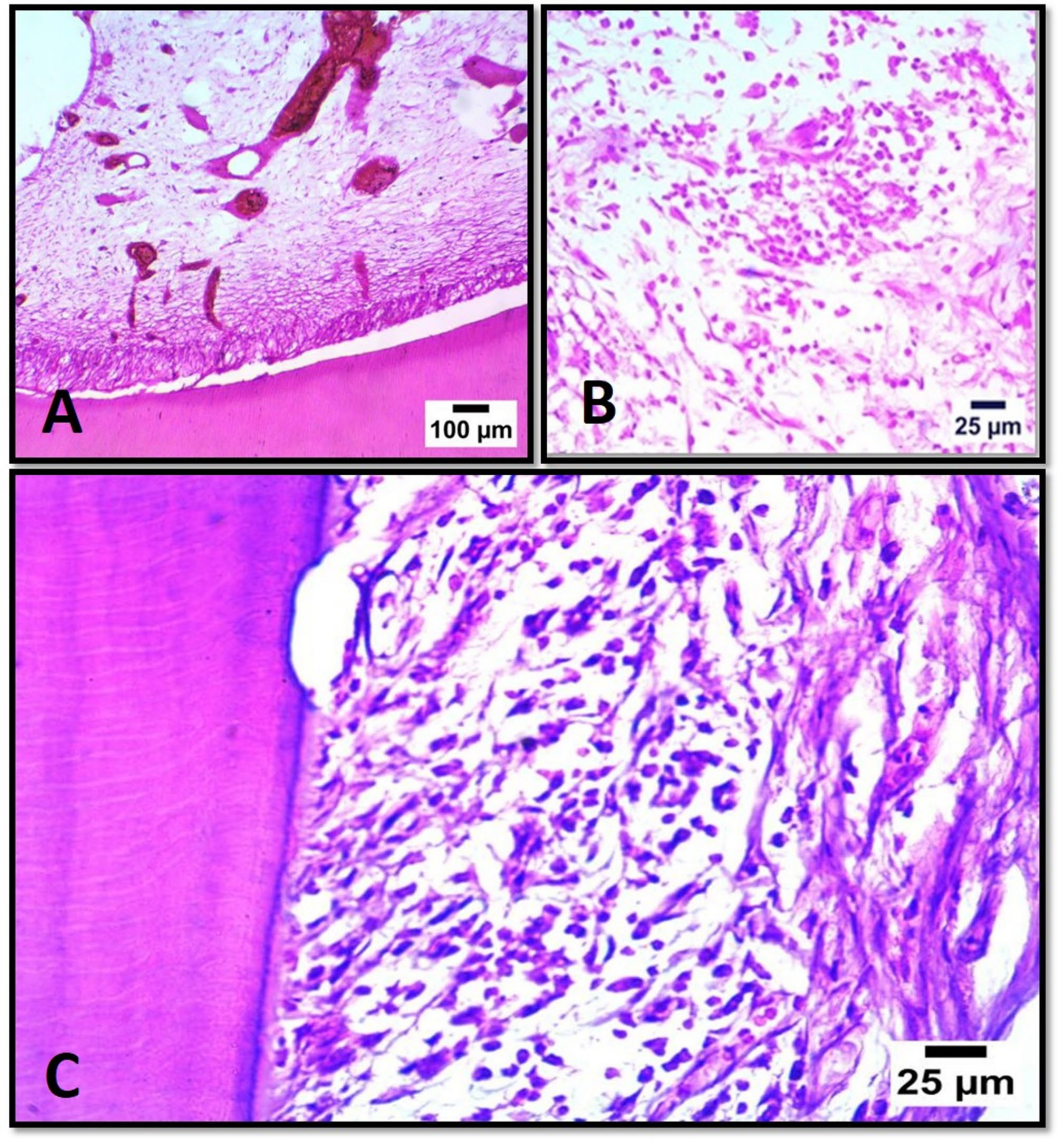
Representative photomicrographs of different subgroups showing noticeable angiogenic activity in subgroup B1 (**A**), noticeable inflammatory cells infiltration in subgroup B2 (**B**), and infiltration of periodontal ligament with high number of mononuclear inflammatory cells in subgroup B2 (**C**). (H&E stain)

There were no statistically significant differences in new hard tissue formation between subgroups A1 and B1 (*P* = 0.150) as well as between subgroups A2 and B2 (*P* = 0.317). Subgroup B2 had the highest mean values, whereas subgroups A1, A2, and B1 had the lowest mean values (00.00). Each subgroup showed varying levels of pulp tissue disorganization, angiogenic activity, and infiltration of inflammatory cells as shown in Figs. 1 and 2.

## Discussion

Direct pulp capping as a vital pulp therapy approach was selected in this study to evaluate the histological reaction of exposed healthy pulp tissue when capped with two DPC agents at two time intervals. This study compared the effects of the Tideglusib GSK-3 inhibitor Alzheimer medication and MTA as DPC agents. Since the current results showed that Tideglusib and MTA differ significantly in terms of the inflammatory cells count and the disorganization of pulp tissue, the study’s null hypothesis was rejected.

The novel biological method, which has been documented in the literature, enables the capping agent to resorb over time to fully restore the lost natural dentine by enhancing the migration of stem cells that reside inside the pulp tissue to induce reparative dentine formation following pulp exposure. Normal dentine volume cannot be achieved because MTA or other capping agents cannot be absorbed, stay stable inside the pulp cavity, and do not break down over time and thus normal dentine volume cannot be restored completely [14].

Since in vitro experiments cannot accurately assess in vivo tissue response, the current study was conducted on animals. To assess the possible hazards of introducing novel dental materials or procedures to the market, animal testing is required [22]. To guarantee complete root development and their resemblance to human teeth in terms of length and size, this study was conducted on adult dog teeth [11, 12]. Dogs are also strong animals that can open their mouth wide, which makes it easier to work on their teeth. Their teeth structure is also appropriate for using them as an experimental model in animal research [5, 6].

To avoid any cofactors that could impede pulp reaction, only intact, healthy teeth with healthy pulps were chosen for this study. When compared to carious exposures following DPC techniques, it is generally accepted that accidental aseptic pulpal exposures yield a more predictable prognosis in maintaining pulp vitality [23].

In order to prevent final restoration failure under mastication forces during the follow-up period, class V cavities were prepared on the buccal surfaces of a few chosen intact teeth. Saline irrigation was used to start physiologic hemostasis and remove dentine chips that might impede pulp healing [1, 7].

Numerous investigations have demonstrated that the Wnt signaling pathway is activated instantly in response to tissue damage and appears to be crucial for tissue healing [24]. When GSK-3 is present and Wnt ligand/receptor binding is not present, this enzyme phosphorylates β-catenin, which causes degradation. The Wnt/β-cat signaling pathway is triggered by the addition of Wnt signaling agonists, such as GSK-3 enzyme inhibitors, which inhibit the GSK-3 enzyme and may offer a useful method of promoting reparative dentine formation, thereby replacing lost dentine with naturally occurring new dentine after decay removal [14]. For DPC, the GSK-3 inhibitor Tideglusib was chosen because, as several studies have demonstrated, it inhibits GSK-3 enzymes and promotes the formation of reparative dentine [14, 25]. To enable full formation of lost natural dentine, the diluted Tideglusib medication was administered to the exposure site using an absorbable collagen sponge [14].

Fibroblasts are the main cell type in pulp tissue and periodontal ligament, and Tideglusib has dose-dependent cytotoxic effects on them; higher doses result in a lower cell viability percentage [26]. Fibroblasts do not proliferate when Tideglusib concentrations are increased from 50 nM/mL to 200 nM/mL, and cytotoxicity increases significantly after 24 h of high concentrations [14, 27]. Because Elturki et al. discovered that the maximum viability percentage of rabbit dental pulp cells at a dosage of 34 nM/mL Tideglusib [28], we utilized 30 nM/mL of Tideglusib in the current investigation.

The MTA was selected as the positive control in this study so that it could be compared to the medication Tideglusib. When used for vital pulp therapy, MTA has been found in numerous studies to have excellent bioactivity, biocompatibility, and sealing ability, resulting in favorable clinical outcomes [1, 5, 7, 9]. MTA causes an alkaline pH and causes growth factors and stem cells to migrate, which causes the formation of a calcific barrier [29]. To reduce any negative effects on the pulp tissue, MTA was packed with a light pressure once hemostasis was achieved [30].

Since the majority of published animal studies in the literature involving vital pulp therapy used time intervals between four and eight weeks [1, 5, 7, 9], the enrolled animals in the current study were evaluated after the follow-up period of three and eight weeks.

Following capping, pulp tissue reaction is primarily determined by material biocompatibility, which is reflected in the degree of inflammation, and bioactivity, which is reflected in the formation of hard tissue at the agent-pulp tissue interface [31]. This study used hematoxylin and eosin staining to perform histopathology assessment regarding the number of inflammatory cells, pulp tissue disorganization, and new hard tissue formation. This is because it is generally accepted in the literature that clinical evaluation of DPC procedures is inadequate due to inflammatory conditions that hinder healing are difficult to diagnose clinically [32].

When compared to Tideglusib after three weeks, MTA produced the lowest mean inflammatory cells count and pulp tissue disorganization, indicating mild to moderate inflammation with superior soft tissue preservation. After eight weeks, MTA revealed improved pulp tissue organization and preservation along with absence of inflammatory cells. In terms of pulp tissue disorganization and the number of inflammatory cells, MTA and Tideglusib demonstrated statistically significant differences during the first and second evaluation periods. Following tissue damage, acute inflammation is a vital reaction that brings inflammatory cells to the site of the injury in order to remove the necrotic tissues. Nevertheless, inflammatory cells can also cause tissue damage by secreting enzymes, harmful oxygen radicals, and chemical mediators that can harm endothelium [33]. Reduced pulp tissue inflammation could be a sign that the DPC materials are biocompatible. As a result, reduced inflammation may be considered a pulp capping agent success [34]. These outcomes are completely consistent with the results of numerous earlier studies which found that MTA, when used as DPC in dogs, exhibited minimal inflammatory responses after 21 days [1, 35]. Furthermore, two months after MTA DPC, inflammatory phenomena vanish [1, 5, 7]. These results could be interpreted as a result of MTA’s superior properties, which include alkaline pH after setting, better marginal adaptation, and minimal cytotoxicity [36, 37]. Additionally, during the second evaluation period, MTA revealed dilated blood vessels, which could be a sign of active dentinogenesis [38]. Tideglusib, on the other hand, demonstrated more inflammatory responses and soft tissue disorganization during both assessment periods. These results could be explained by the collagen sponge’s biodegradation, which weakened the bacterially tight seal underneath the finished restoration. Following DPC, bacterial infiltration has a detrimental effect on the pulp tissue [23]. Thus, after the final restoration is placed, the biostability of the capping materials is thought to be a crucial component in preventing bacterial penetration to the exposure site.

After three weeks of evaluation, neither MTA nor Tideglusib demonstrated any indications of calcific barrier formation; however, after eight weeks, only two MTA specimens displayed partial hard tissue formation (score 1). According to various studies in the literature, the presence of a calcific barrier at the interface between the pulp and the capping material is a contentious issue because the development of a dentinal bridge does not indicate that the pulp tissue is in a healthy state or that an ideal seal has been achieved; rather, it may indicate pulp healing or a response to irritation [23, 39]. The findings of this study contradicted those of another study that assessed the development of reparative dentine in rats following direct capping of exposed pulp with MTA and 50 nM Tideglusib. After four and six weeks of follow-up, the authors concluded that Tideglusib causes denser dentine formation at the injury site than MTA [14]. This difference could be attributed to the different animal model used by Neves et al. who used rats [14]. Rats have wide-apex teeth that grow permanently and a quick biological reaction [40]. Neves *et al.‘s* findings are concerning because, if this level of calcification developed inside the dental pulp in just four weeks, it may indicate that the entire pulp may calcify over time [41].

The present findings are entirely consistent with those of Sukajintanakarn et al., who assessed various DPC materials, including Tideglusib, in a human tooth culture model. The authors concluded that, in contrast to biodentine, 50 nM Tideglusib did not exhibit any mineralization formation in the pulp tissue, which may have been caused by the absence of blood supply in the human tooth culture model [42].

In the present study, absence of new hard tissue formation after three weeks of DPC with MTA are completely consistent with results of previous studies after three weeks in a pig model [19] and four weeks in a dog model [43]. Moreover, only two out of fourteen samples (14.3%) showed partial new hard tissue formation (score 1) following eight weeks of DPC with MTA, according to the current data. These results are in contrast to those of Tziafa et al., who observed that, following an 8-week follow-up period, reparative dentine formed in 85.7% of miniature swine teeth treated with MTA [19]. The disparity in sample sizes, methodology, and the animal model employed in the two investigations may be the cause of this contradiction. In this regard, following two months of DPC with MTA, 20% of dog teeth showed complete hard tissue formation (score 2) [9]. Furthermore, following 12 weeks of DPC with MTA, 87.5% of dog teeth exhibited complete hard tissue formation (score 2) [6].

Because it can increase the proliferation and development of stem cells in the tooth pulp, Tideglusib may offer advantages beyond its conventional role as a GSK-3 inhibitor [44]. Thus, it is proposed that future studies focus on generating novel, unique treatment ways to increase dentin-pulp complex regeneration and enhance clinical results in dental pulp therapy. Further studies are required to assess the cytotoxicity of GSK-3 inhibitors on dental pulp cells. Future clinical studies should also assess the bioactivity and biocompatibility of GSK-3 inhibitors on the healing of exposed crucial pulps. The study’s main limitation is the relatively short observation period, which would not have given enough time for the full creation of a dentin bridge. Future research is therefore advised to evaluate the long-term outcomes of Tideglusib as a DPC agent.

## Conclusion

Tideglusib induces higher soft tissue disorganization and an inflammatory reaction within the pulp cavity than ProRoot white MTA when applied as direct pulp capping materials in a dog model.

## Data Availability

All data used and/or analyzed during this research are available from the corresponding author on reasonable request.
